# Early Detection of Grapevine (*Vitis vinifera*) Downy Mildew (*Peronospora*) and Diurnal Variations Using Thermal Imaging

**DOI:** 10.3390/s22093585

**Published:** 2022-05-08

**Authors:** Bar Cohen, Yael Edan, Asher Levi, Victor Alchanatis

**Affiliations:** 1 Agricultural Research Organization, Agricultural Engineering, P.O. Box 15159, Rishon Le Zion 7528809, Israel; cohenbar16@gmail.com (B.C.); asherl@volcani.agri.gov.il (A.L.); 2 Department of Industrial Engineering and Management, Ben-Gurion University of the Negev, P.O. Box 653, Beer-Sheva 84105, Israel; yael@bgu.ac.il

**Keywords:** precision agriculture, disease detection, pre-symptomatic diagnosis, classification, fungal infection, biotic stress, viticulture

## Abstract

Agricultural industry is facing a serious threat from plant diseases that cause production and economic losses. Early information on disease development can improve disease control using suitable management strategies. This study sought to detect downy mildew (*Peronospora*) on grapevine (*Vitis vinifera*) leaves at early stages of development using thermal imaging technology and to determine the best time during the day for image acquisition. In controlled experiments, 1587 thermal images of grapevines grown in a greenhouse were acquired around midday, before inoculation, 1, 2, 4, 5, 6, and 7 days after an inoculation. In addition, images of healthy and infected leaves were acquired at seven different times during the day between 7:00 a.m. and 4:30 p.m. Leaves were segmented using the active contour algorithm. Twelve features were derived from the leaf mask and from meteorological measurements. Stepwise logistic regression revealed five significant features used in five classification models. Performance was evaluated using K-folds cross-validation. The support vector machine model produced the best classification accuracy of 81.6%, F1 score of 77.5% and area under the curve (AUC) of 0.874. Acquiring images in the morning between 10:40 a.m. and 11:30 a.m. resulted in 80.7% accuracy, 80.5% F1 score, and 0.895 AUC.

## 1. Introduction

Plant diseases are a major cause of production losses and economic losses in the agriculture industry [1]. Pathogens are responsible for direct yield losses of 20–40% of global agricultural productivity [1]. In order to ensure sustainable agriculture, it is essential to monitor plant health to prevent disease spread with as little damage to crop production as possible. However, the main challenge is the difficulty in determining the physical, chemical, and biological changes in plants before symptoms of infection appear [2].

Disease detection techniques can be classified into invasive and non-invasive methods. Invasive techniques involve destructive leaf sampling followed by chemical treatments after direct identification of the pathogen [3]. Non-invasive techniques identify plant diseases by detecting the impact of the pathogen on the physiological plant response. Currently, the most promising non-invasive disease detection methods are sensors that measure temperature, reflectance, or fluorescence [4,5].

Leaf temperature is a rapid response variable that can reveal crop stresses before visible symptoms appear [6]. Stressed plants respond with physiologic protection mechanisms that lead to changes in leaf surface temperature [4]. Infrared thermography (IRT) enables the evaluation of the plant temperature related to changes in water status and transpiration due to infections by pathogens. Temperature differences within individual leaves, plants, and crops indicate the presence of disease in plants. Compared to optical, multispectral, and hyperspectral sensors, thermal sensors have been shown to be more effective at detecting disease-induced early modifications [4]. For example, an infrared (IR) camera was able to differentiate biotic from abiotic stress on cotton [7].

Grapevine downy mildew (DM) is a disease of the foliage caused by Oomycete *Plasmopara viticola* that spreads through extremely efficient asexual reproduction cycles [8]. Originally from North America, DM was accidentally introduced to Europe at the end of the 19th century, where it caused extensive damage to the grape industry [9]. During the spring, oospores germinate to produce macrosporangia, which under wet conditions release zoospores. When rain splashes the zoospores into the canopy, they swim to and through the stomata, where the primary infection occurs. The optimal environmental conditions for primary infection to occur are at least 10 mm of rainfall (or irrigation), and a temperature of at least 10 °C over 24 h [10]. At early stages of infection, DM causes an increase in transpiration rate and a decrease in leaf temperature. The opposite occurs with further DM development: an appearance of chlorotic and necrotic tissue, increased water loss, and an inability of plant tissue to regulate stomatal opening.

The maximum temperature difference (MTD) within a leaf increases during pathogenesis with the formation of necrotic tissue [11]. IRT was used to detect spread of rose DM infections one or two days before the appearance of visible symptoms [12] and cucumber DM before visual symptoms as well [13]. The initial signs of infection in the thermal images were observed as early as four days after infection. However, IRT is often subject to environmental factors such as ambient temperature, sunlight, rainfall, or wind speed [4]. Changes in environmental conditions may affect leaf temperature, making it difficult to differentiate it from a change caused by infection or disease [14]. Alchanatis et al. [15] found that, for estimating and mapping water status variability of cotton, best results from thermal images were achieved at midday (12:00 p.m.–2:00 p.m.).

A previous study showed the feasibility of detecting grapevine DM by support vector machine (SVM) on a limited set of data derived from thermal images [16]. Looking at a small number of infection intervals, DM was detected with an accuracy of 69.2% and F1 score [17] of 74.9%.

The aim of this study was to determine whether thermal imaging can be used to reveal early stages of *P. viticola* infection. The specific objectives were to: (i) extract features for classification based on temperature and image processing algorithms; (ii) develop classification models to distinguish between infected and healthy grapevine leaves; and (iii) determine the best time during the day to acquire thermal images for DM detection.

## 2. Materials and Methods

### 2.1. Plant Material and Experimental Design

Experiments were conducted in six campaigns between the end of December 2019 and the end of October 2020 on 169 grapevine plants, cultivar ‘Chardonnay’. The plants were transplanted into plastic pots with a mixture of organic soil (Even Ari Green LTD., Beit Elazari, Israel), and grown in experimental greenhouses at 25/18 °C (day/night) in Evogene farm, Naan Road, Israel (31°47′ 20.472″ N, 35°12′ 3.888″ E). In each campaign, between 15 to 34 plants were tested (depending on availability) and included different imaging days (healthy leaves and 1, 2, 4, 5, 6, and 7 days after inoculation). Images on the 3rd day after inoculation were not acquired because it fell on non-working days, so it was not possible to acquire images on this day. Moreover, according to the literature, symptoms typically appear on thermal images on the 4th day after inoculation; therefore, the 3rd day data were not completed. Plants received two daily irrigations of fresh water with organic liquid fertilizer (ICL Fertilizers, Dublin, OH, USA). Each of the six campaigns included the following stages:

1. For each stem, the second unfolded leaf from the apex was selected for inoculation and was marked with a color clip or aluminum foil (2–6 leaves in each plant);
2. On the first day of the campaign, images of the healthy leaves were acquired;
3. The leaves were spray-inoculated with $1\times 10^{4}$ concentration of *P. viticola* onto the lower surface using a hand sprayer;
4. The inoculated plants were incubated in a high humidity chamber under optimal environmental conditions in order to allow the pathogen to infect the host tissue and cause DM to develop;
5. In the period of 1–7 days after inoculation, images of healthy and infected leaves were acquired. Images were only acquired on sunny days with a clear sky, meaning that, if weather conditions were not suitable on a particular date, images were not acquired. Table 1 depicts the dates when image acquisition was conducted for each campaign;
6. After the last imaging day (day number 7), the leaves were placed in Petri dishes in order to evaluate the level of the developed disease, which was visually rated by an expert between 0 and 10 (0—healthy, 10—severe disease).

The diurnal response of leaf temperature was measured on 27 October. Images were acquired along the entire day at seven different times (rounds) between 7:00 a.m. and 4:30 p.m. (Table 2). Each round lasted about one and a half hours and included about 87 samples. In each plant, six leaves were sampled: three were six days after inoculation, and three were healthy. Each plant received two daily doses of water: one before the first round and one before the fourth round.

### 2.2. Thermal and RGB Image Acquisition

To allow optimal photosynthesis of the plants, imaging was conducted outside of the greenhouse. The plants were taken out of the controlled greenhouse and placed outdoors for at least one hour before imaging to allow them to adjust to the environmental conditions that were different from the greenhouse conditions. The images were acquired between 10:00 a.m. and 3:00 p.m. to ensure high solar radiation that allowed plants to conduct photosynthesis. Each leaf was placed directly in front of the sun to avoid changes in the illumination of the plant surfaces with the sun angle changes. Meteorological conditions were continuously monitored and included measurements of air temperature (°C), relative humidity, solar radiation (W/m^2^), wind speed (m/s), and wind direction. An image of each leaf was acquired by two cameras—a thermal camera (FLIR SC655, FLIR Systems, Melville, NY, USA) and an RGB camera (Canon EOS6D, Canon Inc., Taby, Sweden) that was used for documentation purposes. The IR camera uses an uncooled microbolometer detector with a resolution of 640 × 480 pixels, sensitive in the spectral range of 7.5–13 μm, possesses an accuracy of ±2 °C or ±2% of the reading, and thermal sensitivity of 0.05 °C @ + 30 °C. For each leaf, the thermal camera acquired a half-minute video and the RGB camera acquired two images. One image from each video was selected for classification. The image was manually selected by visually estimating the maximal leaf surface exposed to the camera.

### 2.3. Datasets

The classification dataset included 1403 records (599 healthy leaves and 804 infected leaves). The records included thermographic measurements of the leaves, meteorological measurements collected simultaneously, calculated features from raw data, and manual evaluation of the disease severity. To determine the earliest day that a model can detect the disease, a subset was created from this dataset, which included records with actual disease severity of 5 or higher. This set included 1097 records (599 healthy leaves and 498 infected leaves). Outliers were removed from the new set, which resulted in 1012 records (571 healthy leaves and 441 infected leaves). The dataset for determining the best acquisition time contained 575 records (280 healthy leaves and 295 infected leaves).

### 2.4. Algorithm for Leaf Delineation

Leaves were segmented using edge detection with the ‘Chan-Vese’ active contour algorithm [18]. This method ensures an unbiased contour enabling to either shrink or expand based on the image features. The software was implemented using MATLAB version R2019b (MathWorks Inc., Natick, MA, USA) with additional functions (Shawn Lankton, 2007). The inputs for the algorithm were a thermal image converted to grayscale and an initial mask; the outputs were an image with the leaf contour and a final mask. For each leaf, the position and size of the initial mask were set manually. The active contour algorithm was run on the mask for a maximum of 100 iterations with a smoothing term of 0.3 (Lambda).

### 2.5. Feature Extraction

Leaf features were calculated from the leaf’s mask (Table 3). The thermal camera provided leaf absolute temperature, after being provided with the values for ambient temperature, reflected temperature, emissivity, and distance from the object. For the analysis, relative values were used by normalizing features expressing temperature by T—T*air*. Healthy leaves had an average temperature of 30 °C with a standard deviation of 4, whereas infected leaves had an average temperature of 31.1 °C with a standard deviation of 5.22. The difference in the temperature between healthy and infected leaves was around 1 °C.

### 2.6. Analysis

Outliers defined in this research as a data point that is 1.5 times the interquartile range above the upper quartile and below the lower quartile (Q1 − (1.5*IQR) or (Q3 + (1.5*IQR)) were removed. The correlation between the predictors was examined by Pearson’s correlation coefficient. To avoid misleading information, the partial correlation coefficient between the predictors was also calculated. Using a correlation coefficient to determine whether there is a numerical relationship between two variables of interest will produce misleading results if there is another, confounding variable numerically related to them both. Therefore, the partial correlation coefficient controlling the confounding variable was used [20]. The correlation between the predictors to the response variable was examined by point-biserial correlation.

To create supervised learning models, different feature selection techniques were used to identify the best set of features (Information Gain, Fisher’s score, Recursive Feature Elimination, and stepwise logistic regression). A stepwise logistic regression [21] resulted in the significant features (*p*-value < 0.05) that provided the best model accuracy (step, stats, RStudio). The stepwise method combined “forward” and “backward” regression. The features selected by the stepwise logistic regression were used in all models. Pearson’s correlation coefficient [22] examined the correlation between the derived features. To give equal importance to each feature and improve the model’s accuracy, quality, and learning rate, the data were normalized using the Z-score [23]. A zero centering of data were performed by subtracting the mean value from each attribute value, then dividing each dimension by its standard deviation. A Min-Max normalization was also used, but it did not significantly improve the results. Normalization and standardization were conducted with MATLAB. In order to evaluate the statistical validity of the best model, statistical tests were conducted between the best model and the second-best model. An F-test was used to test the null hypothesis that the variances of both models are equal. This test was used to determine which *t*-test to use (equal or unequal variance). A *t*-test was used to test the null hypothesis that both models have equal means. A sensitivity analysis was conducted for different distributions of data to explain the different classification results between the days after inoculation.

### 2.7. Classification Models

Five classification models were trained to classify infected and healthy leaves using MATLAB version R2019b (MathWorks Inc., Natick, MA, USA):

- Decision tree—one of the most widely used and practical methods for inference and classification. It has a fast prediction speed and is easy to interpret. This information gain method does not assume any statistical properties of the data itself (e.g., normal distribution) and, as such, it is best suited to this case where the statistical distribution is unknown. When building a decision tree, overfitting may arise, which is represented in the decision tree as a deep tree with many levels. To avoid over-fitting, the maximum number of splits has been limited [24,25].
- Logistic regression—a statistical model that uses a logistic function to model a binary dependent variable and is suitable in this case where there are two classes [26].
- Naïve Bayes (NB)—a statistical classification technique based on Bayes Theorem. A simple supervised learning algorithm which provides fast and accurate classification. The classifier assumes that the effect of a particular feature in a class is independent of other features. However, the algorithm still appears to work well when the independence assumption is not valid [27,28].
- Support vector machine (SVM)—robust prediction models with very high accuracy of disease detection. An SVM training algorithm builds a model that assigns new examples to one category, making it a non-probabilistic binary linear classifier [29,30].
- Ensemble—The technique combines predictions from multiple machine-learning algorithms. In this work, the decision tree ensemble algorithm using the Boosting method was used. Boosting refers to a group of algorithms that trains weak learners sequentially, each trying to correct its predecessor [31].

K-fold cross-validation (K = 5) was used for each model.

### 2.8. Performance Measures

The classification performance was quantified using the accuracy, precision, recall, F1 score [17], and the area under the receiver operating characteristic (ROC) curve, known as the AUC [32]. The class set contained two labels: positive (infected) and negative (healthy). Given a classifier and an instance, there were four possible outcomes:

- True positive (TP): the leaf was infected, and it was classified as infected;
- False-negative (FN): the leaf was infected, but it was classified as healthy;
- True negative (TN): the leaf was healthy, and it was classified as healthy;
- False-positive (FP): the leaf was healthy, but it was classified as

Accuracy is defined as the probability of correctly classifying a test instance:

(1) $$\mathrm{Accuracy}=\frac{\mathrm{TP}+\mathrm{TN}}{\mathrm{Total}\;\mathrm{number}\;\mathrm{of}\;\mathrm{instances}}$$

Precision is called positive predictive value and computed as:

(2) $$\mathrm{Precision}=\frac{\mathrm{TP}}{\mathrm{TP}+\mathrm{FP}}$$

Recall is also referred to true positive rate (TPR) and computed as:

(3) $$\mathrm{Recall}=\frac{\mathrm{TP}}{\mathrm{TP}+\mathrm{FN}}$$

The F1 score is the harmonic mean of the precision and recall and computed as:

(4) $$F1\mathrm{score}=2*\frac{\mathrm{Precision}*\mathrm{Recall}}{\mathrm{Precision}+\mathrm{Recall}}$$

An ROC curve is a graph showing the performance of a classification model at all classification thresholds displaying two parameters:

- Recall (also TPR);
- False Positive Rate (FPR).

False Positive Rate is defined as follows:

(5) $$\mathrm{FPR}=\frac{\mathrm{FP}}{\mathrm{FP}+\mathrm{TN}}$$

An ROC curve plots TPR vs. FPR at different classification thresholds. Lowering the classification threshold classifies more items as positive, thus increasing both False Positives and True Positives. AUC measures the entire two-dimensional area underneath the entire ROC curve.

## 3. Results and Discussion

**Figure 1:**
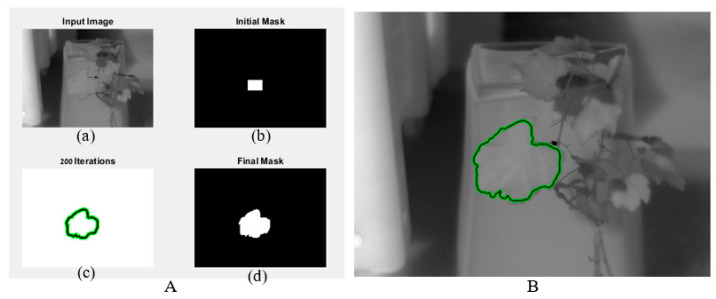
The output of the active contour algorithm. (**A**) includes image, initial, and final mask; (**B**) image with final mask.

Figure 1 depicts the active contour process. Image and initial mask were the algorithm inputs (Figure 1A(a,b)). The algorithm performed iterations to find the contours of the leaf (Figure 1A(c)). After a maximum of 100 iterations, a final mask was obtained (Figure 1A(d)). An image of the leaf with contour was returned (Figure 1B). The returned final mask was a matrix where pixels inside the mask were set to leaf temperature and pixels outside the mask were set to zero.

### 3.1. Classification of Healthy and Infected Leaves

**Figure 2:**
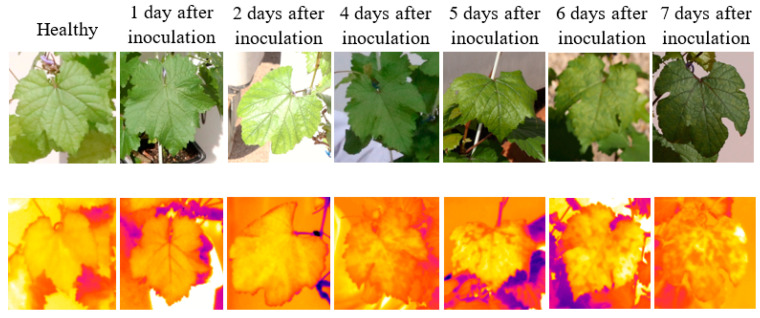
Examples of RGB images (**top row**) and thermal images (**bottom row**) of different leaves from different infected days.

Figure 2 depicts examples of thermal and RGB images acquired at different days after inoculation. The displayed thermal images do not have the same temperature scale to allow higher contrast within each leaf. Compared with the digital images, the changes in the color of the typical thermal images were more noticeable compared to the visual observations.

#### 3.1.1. Feature Selection

The feature selection conducted by stepwise logistic regression (Table 4) resulted in seven significant variables: MTD, STD, CV, T*avg*, median temperature, percentile 90, and CWSI. This result was unexpected because Pearson’s partial correlation matrix showed that T*avg* and median temperature were in a high positive linear correlation (r = 0.97), which led to the expectation that the model would not include both. Another unexpected result was the estimate of T*avg* (β = −9.53) that had the opposite sign of the median temperature (β = 3.696). A possible reason for these results is that the selected method for stepwise logistic regression (“both”) begins with the full model. When the two variables (T*avg* and median temperature) are together in the model and have high multicollinearity and opposite signs, they cancel each other out and stay in the model instead of removing both. Therefore, both variables were removed from the model manually. This led to a higher AIC, resulting with a better model. Finally, to train the classification models, the five remained significant variables were selected: MTD, STD, CV, percentile 90, and CWSI.

#### 3.1.2. Classification Analysis

The hyperparameter values for each model were tuned using Bayesian optimization using MATLAB version R2019b. A Bayesian optimization is an approach that uses the Bayes Theorem to direct the search in each iteration (30 iterations) in order to find the minimum or maximum of an objective function. Table 5 describes the hyperparameters optimized for each type of model, their search range, and their optimal value.

**Figure 3:**
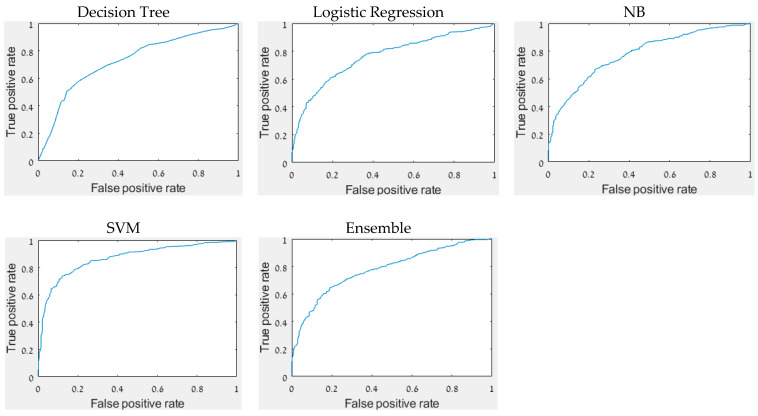
The ROC curve of each model.

The accuracy, precision, recall, F1 score, and AUC of all constructed models (Table 6) reveal that the best results were achieved by the SVM model with major differences between the performance measures of the other models. The accuracy of 81.6% indicates that it is possible to distinguish between infected and non-infected DM on a single grapevine leaf. It is supported by Stoll’s findings, which found statistically significant differences between inoculated and non-inoculated treatments in the slope of the regressions [33]. Figure 3 depicts the ROC curve of each model.

The results of the F-test between the best model (SVM) and the second-best model (NB) using the F1 scores of each of the five folds (cross-validation) revealed that the variances of both models were equal (F (=3.27) < F Critical one-tail (=6.39)) implying that the null hypothesis was not rejected). Thus, the two-tail *t*-test assuming equal variances revealed that the null hypothesis was rejected (t Stat (=−5.56) < t Critical two-tail (=−2.31)). The observed difference between the sample means (0.77–0.67) suggests a significant difference between the models. SVM was chosen to be the best model. To determine the earliest day for DM detection, the results in the following table (Table 7) obtained by the SVM were detailed according to each of the ‘days after inoculation’.

**Figure 4:**
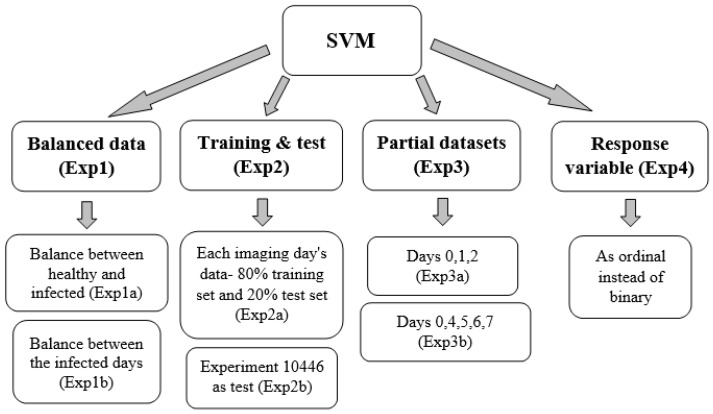
Diagram representing the logical flow of the different analyses.

The results revealed that later days after inoculation produced lower results than earlier ones, contrary to expectations (since the disease develops over time and should be easier to detect as time passes). To determine the cause of this, a series of analyses, as described in Figure 4, were conducted based on different explanations that may have led to these results.

**Figure 5:**
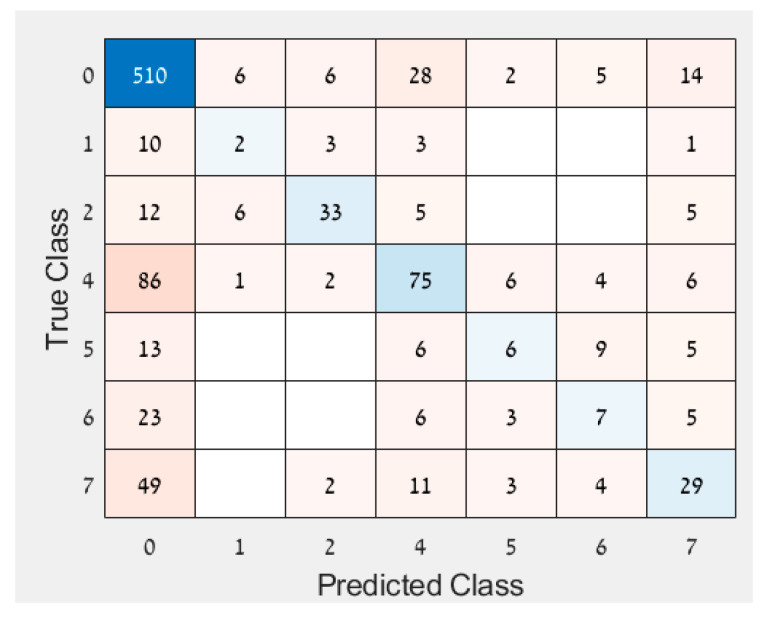
Confusion matrix of ordinal regression.

Exp1 To avoid bias, the dataset was balanced.
  - By removing records from the healthy leaves, the samples number between the diseased and healthy leaves was balanced. The accuracy achieved with these balance results was 79.1%, the F1 score was 77.9%, and the AUC was 0.86. The model’s accuracy of the later days after inoculation improved (Table 8).
  - Besides taking similar numbers of samples from healthy and infected leaves, the number of samples taken each day after inoculation was also balanced. The accuracy achieved was 73.8%, the F1 score was 71%, and the AUC was 0.756. This did not yield improvement in the model’s results, but the accuracy of the later days after inoculation improved greatly.
Exp2 To examine the effects of different climatic conditions on the results, the data were divided into different training and test sets.
  - As the climatic conditions differed between imaging days, it was difficult to classify the data. Each imaging day’s data were split in two: 80% from the data for the training set and 20% from the data for the test set. The accuracy of the training set was 82.5%, with an F1 score of 78.3% and an AUC of 0.886. The test set accuracy was 76.5%, with an F1 score of 70.8% and an AUC of 0.827. Some days’ results improved, while others did not. The model’s performance did not improve.
  - The test set included one specific experiment (No. 10446), and the training set included the rest. Experiment 10,446 included the days 0, 4, 5, 6, 7 after inoculation. Days 5, 6 were not included in any other experiments, so they appear only in the test set. The accuracy of the training set was 86.4%, the F1 score was 82%, and the AUC was 0.892. The test set accuracy was 57.8%, with an F1 score of 48.9% and an AUC of 0.593. The training and test sets were very different in their accuracy. Even on the days that appear in the training set (0, 4, 7), the results are poor. According to this analysis, it is not possible to classify healthy and DM infected from images acquired on an imaging day that is not included in the training set.
Exp3 Considering that the accuracy on days 1 and 2 after inoculation was greater than that of the other days, it was tested whether these days affect the prediction.
  - The following hypothesis was tested: whether with a dataset of leaves from healthy and 1 and 2 days after inoculation, the accuracy of the prediction for these days still remains high (Table 9). The data for this analysis were balanced. The accuracy achieved was 91.9%, the F1 score was 92.1%, and the AUC was 0.961. The results showed that, even without the later days, the early days’ predictions held true very well.
  - The same was done by using a dataset of days 0, 4, 5, 6, and 7 after inoculation. Here also the data were balanced (Table 10). The accuracy achieved was 79.1%, with an F1 score of 78.4% and an AUC of 0.856. The results showed that, when days 1 and 2 after inoculation were removed, the classification results of the later days improved.
Exp4 Since the results of the other approaches varied each day after inoculation, an analysis was performed to examine if the response variable should be ordinal instead of binary. The assumption was that disease development increased every day, suggesting some kind of order as imaging days progressed. Therefore, an ordinal regression was performed on the imbalanced data. A new feature selection was conducted to accommodate for the new response variable and resulted in the following features: MTD, IQR, MAD, median, perc10, perc90, and CV. The accuracy of the ordinal classification was 65.4% with many observations classified as healthy even though they were infected (and were not classified as infected on another day, Figure 5). Since it does not matter which day after inoculation was classified, but whether the leaf was healthy or infected, the results were converted to binary so all days that were not classified as 0 (healthy) were deemed infected. This classification of the converted response variable resulted in an accuracy of 74.9%. The binary response variable performed better than both the ordinal response variable and the converted response variable and hence was selected.

All approaches were also evaluated after a new feature selection process was performed (except for Exp4, which anyway included a new feature selection). The purpose of this was to examine whether the results and features selected were different. In each approach, the new selected features were very similar to the first selection, with performance differing by only +−3%. These results indicated that the first feature selection was indeed suitable for the different datasets.

Table 11 summarizes the results of the different approaches used to explain the differences in disease detection between days after inoculation. Although the best results were with the SVM model using a dataset with healthy and infected leaves from only 1 and 2 days after inoculation, this dataset itself was not selected since it included only the two first days after inoculation without the remaining days. These good results were probably due to the fact that the plants were disturbed by the controlled inoculation, which was what was probably actually detected. The best relevant results were obtained from an SVM model using a balanced dataset of healthy leaves and infected leaves.

A possible explanation for the differences in disease detection between days after inoculation (days 4–7 after inoculation) might be that IRT measurements are influenced by many other factors that affect leaf temperature, including ambient temperature, humidity, sunlight, and wind [34]. The images were acquired at different dates, times of day, and under different environmental conditions, which may have affected the characteristics of the images or even the plants. Although each variable related to temperature has been normalized (T-T*air*), this might not have been sufficient to accommodate environmental changes.

The classification results of days one and two after inoculation, which were better than those of the other days, was probably not because the DM was detected early; it is more likely that the inoculation itself, which was manually induced, may have affected the leaves locally and caused a strong physiological reaction to the leaves. As the virus penetrated the plant, the local effect diminished.

Since each of the models (the model with data of days 0, 1, 2 and the model with data of days 0, 4, 5, 6, 7) provided good results, future research should consider dividing the data into three groups: healthy, infected at first days, and infected at late days.

### 3.2. Best Acquisition Time

**Figure 6:**
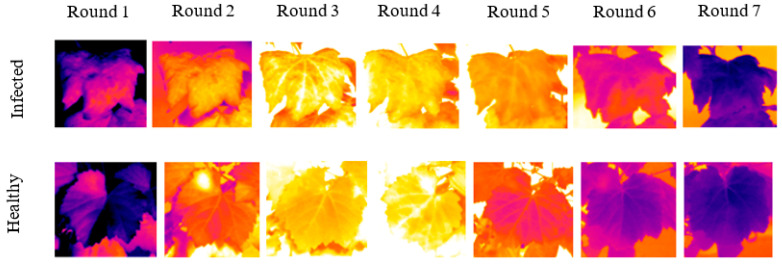
Thermal images from infected and healthy leaves acquired at different times of the day.

Examples of thermal images from infected and healthy leaves acquired at different times of the day are depicted in Figure 6. The displayed images have the same scale (23.6°–34.6 °C).

For all rounds, the accuracy, F1 score, and AUC obtained by the SVM model are shown in Table 12. Best results were obtained when data were collected 10:40 a.m.–11:30 a.m. (round 3) with accuracy differences of about 6% and 23% from the next best and worst rounds, respectively. These results are different from results of Alchanatis et al. [15], who found that midday (12:00 p.m.–2:00 p.m.) was the best time to map and estimate water status variability using thermal imaging. Although these are two different problems, they are similar in expectance to see temperature differences within the leaves.

The results of the F-test between the best round (3) and the second-best round (1) using the accuracy of each of the five folds from cross-validation (since the data were balanced) revealed that the null hypothesis was not rejected (F (=4.08) < F Critical one-tail (=6.39)), implying that the variances of both rounds were equal. Thus, the two-tail *t*-test assuming equal variances revealed that the null hypothesis was not rejected (-t Critical two-tail (=−2.31) < t Stat (=−1.12) < t Critical two-tail (=2.31)). The observed difference between the sample means (0.807–0.751) is not convincing enough to say that the results differ significantly.

Although the 3rd round was not statistically better, records from these hours were analyzed to generalize the findings since best results were obtained at this acquisition time. A special new dataset of 239 records (95 healthy and 144 infected) was created using only observations that were acquired from 10:40 a.m. and 11:30 a.m. from the classification model (the complete dataset). This dataset was used to create a new model; the results are detailed in Table 12. To improve the results, a new feature selection was performed. The selected features were: MTD, T*avg*, T*min*, perc10, perc90, and CV. The new features improved the results and the model produced good results (Table 13).

As compared with the model that used all the data, the model from the best hours included fewer observations, but the results were quite similar. This model would likely perform even better if it were trained on a larger dataset. Therefore, it is worth considering acquiring images at similar times during the day, even if they are acquired on different days. Further research is needed on this topic. However, even when the acquisition occurred during the best hours, results are strongly affected by the environmental conditions, which may change day by day, and impact the results.

## 4. Summary and Conclusions

The results indicate that thermograms can detect downy mildew, even before any visible symptoms appear. The best model for classifying between healthy and infected leaves was an SVM model built on a balanced dataset with the following features: MTD, STD, percentile 90, CV, and CWSI. The model resulted in 10% higher performance than all other models tested (81.6% accuracy, 77.5% F1 score, and 0.874 AUC). The inconsistent results between the days (days 4–7 after inoculation) could not be explained.

The best time of day for acquiring images for downy mildew detection was between 10:40 a.m. and 11:30 a.m. resulting in 80.7% accuracy, 80.5% F1 score, and 0.895 AUC. Using images from the best hours probably can improve performance, even if the images are not from the same days. However, even when the image acquisition is conducted at the best time, variations in illumination cannot be avoided, resulting in reduced performance. There is a trade-off between using a large and wide database (acquired along many dates) and detecting the disease (the fewer dates, the easier). Early disease detection using thermal imaging is possible and should be further advanced.

## Figures and Tables

**Table 1:** The experimental schedule.

Campaign	Date	Days after Inoculation	Number of Infected Leaf Samples	Number of Healthy Leaf Samples
1	30 December 2019	1	71	17
1	31 December 2019	2	74	-
2	16 January 2020	4	60	-
3	26 January 2020	7	52	-
4	3 March 2020	2	94	85
4	5 March 2020	4	86	-
4	8 March 2020	7	86	-
5	26 March 2020	0	-	323
5	2 April 2020	4	101	-
6	25 October 2020	4	45	45
6	26 October 2020	5	45	45
6	27 October 2020	6	45	43
6	28 October 2020	7	45	41

**Table 2:** Diurnal acquisition: Time of image acquisition and number of samples.

Round Number	Acquisition Time	Number of Samples
1	7:15–8:25	88
2	9:00–9:45	88
3	10:40–11:30	88
4	12:25–13:15	87
5	14:15–15:05	86
6	15:20–16:00	86
7	16:00–16:30	52

**Table 3:** Features description.

Variable Name	Description	Range	Symbol	Calculation
Minimum temperature	The minimum temperature in the leaf, minus the air temperature measured at the same time	(−6.3)–10.7	Tmin	Tmin-Tair
Maximum temperature	The maximum temperature in the leaf, minus the air temperature measured at the same time	(−4.2)–14.6	Tmax	Tmax-Tair
Average temperature	The average of the leaf temperatures values, minus the air temperature measured at the same time	(−5.11)–12.98	Tavg	Tavg-Tair
Median temperature	The median of the leaf temperatures values, minus the air temperature measured at the same time	(−5.06)–13.11	median	median-Tair
Maximum temperature difference	The difference between the maximum and minimum temperature in the leaf	0.5–7.1	MTD	Tmax-Tmin
Standard deviation	The standard deviation value of the leaf temperature values	0.1–1.73	STD	std
Interquartile range	A measure of statistical dispersion and equal to the difference between 75th and 25th percentiles	0.17–3.28	IQR	T0.75-T0.25
Mean absolute deviation	A robust measure of the variability. Defined as the mean of the absolute deviations from the mean of the data	0.1–1.53	MAD	$\frac{\sum \left|T_{i}-mean\right|}{n}$
Coefficient of variation	Or relative standard deviation, a standardized measure of the dispersion of a probability distribution or frequency distribution.	0.004–0.061	CV	$\frac{STD}{mean}$
Percentile 10	The percentile is a score at or below which a given percentage fall, minus the air temperature measured	(−5.9)–11.9	perc10	T0.1-Tair
Percentile 90		(−4.8)–13.9	perc90	T0.9-Tair
Crop water stress index	A means of irrigation scheduling and crop water stress quantification based on leaf temperature measurements and prevailing meteorological conditions [19]	0.37–1.53	CWSI	$\frac{T_{l}-T_{wet}}{T_{dry}-T_{wet}}$

**Table 4:** Stepwise regression-estimated coefficients, standard errors, and *p*-value.

Variable	Estimated Coefficients	Standard Errors	*p*-Value
MTD	0.6543	0.1782	0.00024
STD	−2.4373	1.1323	0.03136
CV	79.2226	24.7641	0.00138
percentile 90	0.2709	0.0388	3.06 × 10^−12^
CWSI	1.6405	0.4575	0.00034

**Table 5:** Hyperparameters, search range, and selected optimal value.

Model	Hyperparameter	Range	Optimal
Decision Tree	Maximum number of splits	[1, 1011]	17
	Split criterion	Gini’s diversity index, Twoing rule, and Maximum deviance reduction	Maximum deviance reduction
Naive Bayes	Distribution names	Gaussian and Kernel	Kernel
	Kernel type	Gaussian, Box, Epanechnikov, and Triangle	Box
SVM	Kernel function	Gaussian, Linear, Quadratic, and Cubic	Cubic
	Box constraint level	[0.001, 1000]	1
Ensemble	Ensemble method	AdaBoost, RUSBoost, LogitBoost, GentleBoost, and Bag	GentleBoost
	Maximum number of splits	[1, 1011]	960
	Number of learners	[10, 500]	498
	Learning rate	[0.001, 1]	0.057385

**Table 6:** All results from all models based on all data.

Model Measure	Decision Tree	Logistic Regression	NB	SVM	Ensemble
F1 score	60.5%	64.9%	66.9%	77.5%	66.7%
Precision	70.5%	70.8%	70.4%	83.1%	69.3%
Recall	53.1%	59.9%	64.2%	71.6%	64.4%
AUC	0.728	0.762	0.782	0.874	0.782
Accuracy	69.9%	71.7%	72.6%	81.6%	72%

**Table 7:** Results by day after infection.

Days after Inoculation	Number of Samples	Number of Misses	Accuracy
0	571	65	88.6%
1	19	2	89.5%
2	61	3	95.1%
4	180	55	69.4%
5	39	4	89.7%
6	44	17	61.4%
7	98	40	59.2%
Model	-	-	81.6%

**Table 8:** Results after balancing healthy and infected samples (Exp1a).

Days after Inoculation	Number of Samples	Number of Misses	Accuracy
0	441	68	84.6%
1	19	1	94.7%
2	61	5	91.8%
4	180	55	69.4%
5	39	5	87.2%
6	44	14	68.2%
7	98	36	63.3%
Model	-	-	79.1%

**Table 9:** Results of a dataset with days 0, 1, 2 after inoculation (Exp3a).

Days after Inoculation	Number of Samples	Number of Misses	Accuracy
0	99	10	89.9%
1	21	1	95.2%
2	78	5	93.6%
Model	-	-	91.9%

**Table 10:** Results of a dataset with days 0, 4, 5, 6, 7 after inoculation (Exp3b).

Days after Inoculation	Number of Samples	Number of Misses	Accuracy
0	399	72	81.9%
4	197	54	72.6%
5	45	5	88.9%
6	45	9	80%
7	112	27	75.9%
Model	-	-	79.1%

**Table 11:** A summary of all approaches and their results.

Approach	F1 Score	AUC	Accuracy
SVM—all data	77.5%	0.874	81.6%
Balance between healthy and infected (Exp1a)	77.9%	0.86	79.1%
Balance between the infected days (Exp1b)	71%	0.756	73.8%
Each imaging day’s data—80% training set and 20% test set (Exp2a)	70.8%	0.827	76.5%
Experiment 10446 as test (Exp2b)	48.9%	0.593	57.8%
Days 0,1,2 (Exp3a)	92.1%	0.961	91.9%
Days 0,4,5,6,7 (Exp3b)	78.4%	0.856	79.1%
As ordinal instead of binary (Exp4)	-	-	74.9%

**Table 12:** Performance for each round of the diurnal measurements.

Measure			
Round No./Time	Accuracy	F1 Score	AUC
(1) 7:15–8:25	75%	75.6%	0.774
(2) 9:00–9:45	72.7%	72.7%	0.794
(3) 10:40–11:30	80.7%	80.5%	0.895
(4) 12:25–13:15	59.8%	61.5%	0.676
(5) 14:15–15:05	65.1%	67.4%	0.691
(6) 15:20–16:00	58.1%	61.7%	0.644
(7) 16:00–16:30	57.7%	59.3%	0.557

**Table 13:** Results of a dataset from 10:40 a.m. to 11:30 a.m. and after a new feature selection.

Model	Number of Samples	F1 score	AUC	Accuracy
Hours 10:40–11:30	239	72.7%	0.764	67.4%
New features	239	80.8%	0.826	76.6%

## Data Availability

The datasets generated and analyzed during the current study are available in GitHub: Data sets (Excel): https://github.com/BarCohenBGU/database.git (29 September 2021). ‘All data new’—the classification dataset included 1403 records. ‘normalized_by_severity’—included 1097 records with actual disease severity of 5 or higher. ‘features_without_outlires’—included 1012 records after removing outlie. Daily dataset (Excel): https://github.com/BarCohenBGU/daily-dataset.git (31 October 2021).

## References

[1] Savary S., Ficke A., Aubertot J.N., Hollier C. (2012). Crop Losses Due to Diseases and Their Implications for Global Food Production Losses and Food Security. Food Secur..

[2] Cui S., Ling P., Zhu H., Keener H.M. (2018). Plant Pest Detection Using an Artificial Nose System: A Review. Sensors.

[3] Sankaran S., Mishra A., Ehsani R., Davis C. (2010). A Review of Advanced Techniques for Detecting Plant Diseases. Comput. Electron. Agric..

[4] Mahlein A.K. (2016). Plant Disease Detection by Imaging Sensors- Parallels and Specific Demands for Precision Agriculture and Plant Phenotyping. Plant Dis..

[5] Mulla D.J. (2013). Twenty Five Years of Remote Sensing in Precision Agriculture: Key Advances and Remaining. Knowl. Gaps. Biosyst. Eng..

[6] Khanal S., Fulton J., Shearer S. (2017). An Overview of Current and Potential Applications of Thermal Remote Sensing in Precision Agriculture. Comput. Electron. Agric..

[7] Falkenberg N.R., Piccinni G., Cothren J.T., Leskovar D.I., Rush C.M. (2007). Remote Sensing of Biotic and Abiotic Stress for Irrigation Management of Cotton. Agric. Water Manag..

[8] Kiefer B., Riemann M., Büche C., Kassemeyer H.H., Nick P. (2002). The Host Guides Morphogenesis and Stomatal Targeting in the Grapevine Pathogen Plasmopara Viticola. Planta.

[9] Gessler C., Pertot I., Perazzolli M. (2011). Plasmopara Viticola: A Review of Knowledge on Downy Mildew of Grapevine and Effective Disease Management. Phytopathol. Mediterr..

[10] Kennelly M.M., Gadoury D.M., Wilcox W.F., Magarey P.A., Seem R.C. (2007). Primary Infection, Lesion Productivity, and Survival of Sporangia in the Grapevine Downy Mildew Pathogen Plasmopara Viticola. Phytopathology.

[11] Calderón R., Montes-Borrego M., Landa B.B., Navas-Cortés J.A., Zarco-Tejada P.J. (2014). Detection of Downy Mildew of Opium Poppy Using High-Resolution Multi-Spectral and Thermal Imagery Acquired with an Unmanned Aerial Vehicle. Precis. Agric..

[12] Caro S.G. (2014). Infection and Spread of Peronospora Sparsa on Rosa Sp.(Berk.)—A Microscopic and a Thermographic Approach. Ph.D. Thesis.

[13] Wen D.M., Chen M.X., Zhao L., Ji T., Li M., Yang X.T. (2019). Use of Thermal Imaging and Fourier Transform Infrared Spectroscopy for the Pre-Symptomatic Detection of Cucumber Downy Mildew. Eur. J. Plant Pathol..

[14] Grant O.M., Chaves M.M., Jones H.G. (2006). Optimizing Thermal Imaging as a Technique for Detecting Stomatal Closure Induced by Drought Stress under Greenhouse Conditions. Physiol. Plant..

[15] Alchanatis V., Cohen Y., Cohen S., Moller M., Sprinstin M., Meron M., Tsipris J., Saranga Y., Sela E. (2010). Evaluation of Different Approaches for Estimating and Mapping Crop Water Status in Cotton with Thermal Imaging. Precis. Agric..

[16] Cohen B., Edan Y., Levi A., Alchanatis V. (2021). Early detection of grapevine downy mildew using thermal imaging. Precis. Agric..

[17] Chinchor N. (1992). MUC-4 Evaluation Metrics. Proceedings of the 4th Message Understanding Conference MUC 1992.

[18] Chan T.F., Vese L.A. (2001). Active Contours without Edges. IEEE Trans. Image Process..

[19] Jones H.G. (1999). Use of Infrared Thermometry for Estimation of Stomatal Conductance as a Possible Aid to Irrigation Scheduling. Agric. For. Meteorol..

[20] Frank K.A. (2000). Impact of a Confounding Variable on a Regression Coefficient. Sociol. Methods Res..

[21] Lemeshow S., Hosmer D.W. (1982). A Review of Goodness of Fit Statistics for Use in the Development of Logistic Regression Models. Am. J. Epidemiol..

[22] Taylor R. (1990). Interpretation of the Correlation Coefficient: A Basic Review. J. Diagnostic Med. Sonogr..

[23] Dhaka V.S., Meena S.V., Rani G., Sinwar D., Kavita, Ijaz M.F., Woźniak M. (2021). A Survey of Deep Convolutional Neural Networks Applied for Prediction of Plant Leaf Diseases. Sensors.

[24] Wang T., Li Z., Yan Y., Chen H. (2007). A Survey of Fuzzy Decision Tree Classifier Methodology. Adv. Soft Comput..

[25] Myles A.J., Feudale R.N., Liu Y., Woody N.A., Brown S.D. (2004). An Introduction to Decision Tree Modeling. J. Chemom..

[26] Scott A.J., Hosmer D.W., Lemeshow S. (1991). Applied Logistic Regression. Biometrics.

[27] Zhang H. (2005). The Optimality of Naive Bayes. Int. J. Pattern Recognit. Artif. Intell..

[28] Rish I. (2001). An Empirical Study of the Naive Bayes Classifie. Phys. Chem. Chem. Phys..

[29] Amari S., Wu S. (1999). Improving Support Vector Machine Classifiers by Modifying Kernel Functions. Neural Netw..

[30] Noble W.S. (2006). What Is a Support Vector Machine?. Nat. Biotechnol..

[31] Rokach L. (2010). Ensemble-Based Classifiers. Artif. Intell. Rev..

[32] Myerson J., Green L., Warusawitharana M. (2001). Area Under the Curve As a Measure of Discounting. J. Exp. Anal. Behav..

[33] Stoll H.R.M., Baecker S.G., Berkelmann-Loehnertz B. (2008). Early pathogen detection under different water status and the assessment of spray application in vineyards through the use of thermal imagery. Precis. Agric..

[34] Granum E., Pérez-Bueno M.L., Calderón C.E., Ramos C., de Vicente A., Cazorla F.M., Barón M. (2015). Metabolic Responses of Avocado Plants to Stress Induced by Rosellinia Necatrix Analysed by Fluorescence and Thermal Imaging. Eur. J. Plant Pathol..

